# Correlation of miR-24-3p and miR-595 expression with *CCL3, CCL4, IL1-beta, TNFalphaIP3,* and *NF-kappaBIalpha* genes in PBMCs of patients with coronary artery disease

**DOI:** 10.17179/excli2022-5266

**Published:** 2022-09-08

**Authors:** Shakiba Kazemian, Reza Ahmadi, Gordon A. Ferns, Ali Rafiei, Fatemeh Azadegan-Dehkordi, Arsalan Khaledifar, Mina Mohammad-Rezaei, Nader Bagheri

**Affiliations:** 1 Student Research Committee, Shahrekord University of Medical Sciences, Shahrekord, Iran; 2 Clinical Biochemistry Research Center, Basic Health Sciences Institute, Shahrekord University of Medical Sciences, Shahrekord, Iran; 3 Brighton & Sussex Medical School, Division of Medical Education, Falmer, Brighton, Sussex, UK; 4 Medical Plants Research Center, Basic Health Sciences Institute, Shahrekord University of Medical Sciences, Shahrekord, Iran; 5 Cellular and Molecular Research Center, Basic Health Sciences Institute, Shahrekord University of Medical Sciences, Shahrekord, Iran; 6 Department of Cardiology, School of Medicine, Shahrekord University of Medical Sciences, Shahrekord, Iran; 7 Department of Immunology, School of Medicine, Iran University of Medical Sciences, Tehran, Iran; 8 Department of Microbiology and Immunology, Faculty of Medicine, Shahrekord University of Medical Sciences, Shahrekord, Iran

**Keywords:** atherosclerosis, cardiovascular diseases, inflammation, miR-24-3p, miR-595, NF-kappaBIalpha

## Abstract

Inflammation has been well recognized to play an important role in developing coronary artery disease (CAD). By regulating essential genes in this pathway post-transcriptionally, MicroRNAs (miRNAs) may help or hinder the development of atherosclerotic lesions. The aim of this study was to investigate the expression of miR-24-3p, miR-595, *CCL3*, *CCL4*, *IL-1β*, *TNFαIP3*, and *NF-κBIα* in the peripheral blood mononuclear cells (PBMCs) of CAD and control groups and to examine whether any correlation exists between the expression of miRs and genes in CAD group. A total of 168 subjects (84 CAD subjects and 84 control subjects) were examined in this research. Expression levels of miR-24-3p, miR-595, CCL3, CCL4, IL-1β*, TNFαIP3*, and *NF-κBIα* in PBMCs were measured using the real-time PCR technique. A comparison of the CAD group with the control group indicated significantly increased expression levels of *CCL3*, *CCL4,* and *IL-1β *(Fold Change (FC)=4, *P*=0.009; FC=2.9, *P*=0.01; FC=1.8, *P*=0.019, respectively) and remarkably reduced expression levels of *TNFαIP3* and *NF-κBIα *(FC=-1.4, *P*=0.03 and FC=-5.9, *P*=0.001, respectively). Moreover, the expression levels of miR-24-3p downregulated (FC=-2.5, *P*=0.005) and miR-595 upregulated (FC=1.9, *P*=0.009) in the CAD group. There was a statistical correlation between the number of clogged arteries with expression levels of miR-24-3p, miR-595, *CCL3*, *CCL4*, *IL-1β*, *TNFαIP3*, and *NF-κBIα* in the CAD group. Also, there was a statistical correlation between expression levels of miR-24-3p and miR-595 with *CCL3*, *CCL4*, *IL-1β*, *TNFαIP3*, and *NF-κBIα* gene expression in the CAD group. In CAD patients, decreased expression of miR-24-3p and increased expression of miR-595 may aid the progression of atherosclerotic plaques by regulating *CCL3*, *CCL4*, *IL-1β*, *TNFαIP3*, and *NF-κBIα* gene expression.

## Introduction

Cardiovascular disease (CVD) is the most significant contributor to mortality and morbidity worldwide. The World Health Organization (WHO) estimates that CVD is accounting for approximately 17.6 million deaths or 31 % of all global deaths per year, of which 80 % occur in low- and middle-income countries and 85 % are due to heart attacks and strokes (McAloon et al., 2016[26]). Among CVDs, CAD is considered an effective form with almost epidemic proportions in most communities and is responsible for more deaths than any other group (Alizadehsani et al., 2018[1]). The primary cause of CAD is atherosclerosis. Atherosclerosis has a number of risk factors, such as obesity, age, smoking, hypertension, inflammation, and diabetes mellitus (Lonardo et al., 2018[24]; Kazemian et al., 2022[20]). Vascular inflammation plays an important role in the pathogenesis of atherosclerosis, and its bidirectional association with lipid accumulation leads to vascular endothelial cell damage and plaque formation (Rafiei et al., 2022[31]; Mohammad-Rezaei et al., 2021[28][27]). CAD is a disease that develops slowly and has minimal symptoms in its early stages. When prevention is possible, non-invasive methods for determining the severity of CAD are critical to patient management. Circulating miRs are therefore interesting non-invasive indicators (Liu et al., 2016[22]). MiRNAs are 20-24 nucleotide long RNA molecules with only one strand attached to target genes' mRNA and controls their expression. Increased levels of miRs are strongly associated with increased risk of a wide range of diseases, including CAD (Bushati and Cohen, 2007[2]; Gangwar et al., 2018[11]; Rafiei et al., 2021[32]). MiRNA levels have been discovered to be tissue-specific and intimately correlated to physiological and pathological changes in the cardiovascular system (Creemers et al., 2012[5]). Furthermore, miRs identified in body fluids are persistent and resistant to digestion by endogenous RNases due to their small size. These properties make miRs suitable biomarkers for CVD (Gilad et al., 2008[13]).

In recent years there have been numerous studies on the potential role of miRs as diagnostic biomarkers in the field of CVD. For example, miR-24-3p inhibits smooth muscle cell (SMC) proliferation and migration in human arteries (Zhu et al., 2015[40]). MiR-595 has also been discovered to be downregulated in immune-related diseases, inhibiting cell proliferation, migration, and invasion *in vitro*. The upregulation of miR-595 expression also inhibits the nuclear factor kappa B (NF-κB) signaling pathway (Wang et al., 2020[38]). NF-κB is a transcription factor that regulates cell differentiation, activation, proliferation, apoptosis, and the development of inflammatory mediators by translating inflammatory stimuli from the environment into gene expression patterns (Queiro et al., 2021[30]). NF-κBIα is one member of the inhibitor of NF-κB (IκB) class which is a part of a negative feedback loop that prevents excessive and irreversible NF-κB activation by retracting it from the nucleus back into the cytoplasm and it is also activated in inflammatory conditions, especially in macrophages and endothelial cells (Laberge et al., 2005[21]; Vallabhapurapu and Karin, 2009[36]). *TNFαIP3 (A20)* gene is a key regulator of inflammation that encodes a broadly expressed cytoplasmic protein that results in inhibition of regulatory genes *TNFα *and *IL-1β *and termination of *NF-κB* (Wolfrum et al., 2007[39]). *IL-1β* is another important modulator of inflammatory pathways which is part of a cluster of genes on chromosome 2 coding for a family of IL-1 proteins (Maruyama et al., 2005[25]).

It is well documented that inflammatory cytokines and chemokines play significant roles in atherosclerosis, pathogenesis and complications (Hopkins, 2013[19]). Inflammatory chemokines CCL3 and CCL4 are of great importance in the production of inflammatory signals and transmitting leukocytes to damaged tissue (Versteylen et al., 2016[37]). CCL3 appears to be primarily derived from ischemia and may play a role in post-ischemia inflammation, probably through neutrophil-induced monocyte recruitment (de Jager et al., 2008[8]).

Therefore, this study aimed to investigate the expression of miR-24-3p, miR-595, *CCL3*, *CCL4*, *IL-1β*, *TNFαIP3*, and *NF-κBIα* in the PBMCs of CAD and control groups and to examine whether any correlation exists between the expression of miRs and genes in CAD group. The combination of the two miRNAs may be more efficacious than either miRNA alone for the diagnosis of CAD.

## Materials and Methods

### Study participants 

A total of 168 Iranian participants who visited the cardiac health clinic with some symptoms like shortness of breath and chest pain were included in the study. All participants were new cases and none of them used any related medications before. Patients who suffered from any of the following conditions were excluded: diabetes, liver disease, kidney disease, chronic infections, autoimmune diseases, and cancer. All participants undergone coronary venous angiography at Hajar Hospital in Shahrekord, Chaharmahal and Bakhtiari Province, Iran. The study was conducted in compliance with the Helsinki declaration, and all participants gave their informed written consent.

### Anthropometric data collection

The researcher also made a note of the patients' demographic details, such as weight, height, body mass index (BMI), systolic and diastolic blood pressure (SBP and DBP), and family background. The SBP and DBP were measured using a regular sphygmomanometer at the beginning of the test and after 15 minutes of sitting.

### Coronary angiography

All participants had angiographic indices (shortness of breath, chest pain, disturbance in exercise test, and echography). Therefore, a cardiologist used angiography to make a definitive diagnosis of CAD. Based on the result of this angiography, participants were divided into 2 groups: Those with 50 % or greater arterial stenosis in one or more of the main coronary arteries, as CAD group, and those who had arterial stenosis of 30 % or less, as control group. Also, coronary angiograms in CAD group were scored according to the vessel score and Gensini score (Gensini, 1983[12]). The vessel score is the number of vessels with significant stenosis (50 % or greater reduction in lumen diameter), ranging from 0 to 3 as CS (Cardiac Stenosis) 1, 2, and 3 (Gensini, 1983[12]).

### Blood sample collection and serum separation

Ten mL of venous blood was drawn from each participant, with 5 mL going into a PBMC separation tube with ethylenediaminetetraacetic acid (EDTA) and 5 mL going into a serum separation tube with coagulation factors. Afterward, serum samples were held at -80°C for further analysis.

### Laboratory measurements 

Biochemical parameters, such as triglyceride (TG), total cholesterol (TC), high-density lipoprotein cholesterol (HDL-C), low-density lipoprotein cholesterol (LDL-C), fasting blood sugar (FBS), creatinine, blood urea nitrogen (BUN), potassium, sodium, and creatine phosphokinase myocardial band (CPK-MB) were measured based on enzymatic and spectrophotometric standards and using the Pars Azmoun Company's kits.

### PBMC isolation and RNA extraction

The PBMC separation was done in a sterile setting (Corkum et al., 2015[4]). Phosphate buffer solution (PBS) was used to dilute the blood (1:1). Then, using Lympholyte (Cedarlane, Ontario, Canada), the blood was centrifuged, and PBMCs were separated using density-gradient centrifugation, following the working method of the tool. Afterward, PBMCs were washed using PBS (2×) and kept at -80 °C. According to the manufacturer's instructions, the total RNA was extracted using RNX-Plus Solution (SinaClon, Iran). Thermo ScientificTM NanoDrop 2000 was used to determine the RNA sample concentration. In order to assess RNA purity and concentration, the 260/280 and 260/230 ratios were calculated for each sample.

### cDNA synthesis and mRNA/miRNA expression 

The RevertAid first strand cDNA synthesis kit (Thermo Scientific, K1622) was used for total RNA, and the BONmiR high-sensitivity miR first-strand cDNA synthesis kit was used for *miRNAs cDNA *synthesis. A Rotor-Gene RG-3000 was used to perform quantitative real-time polymerase chain reaction (real-time PCR) (Corbett Research, Sydney, Australia). The expression levels of *CCL3*, *CCL4*, *IL-1β*, *TNFαIP3*, and *NF-κBIα* mRNA were compared to glyceraldehyde 3-phosphate dehydrogenase (GAPDH) mRNA using the 2^−ΔΔCt^ method. The expression level of miR-24-3p and miR-595 concerning U6 snRNA was measured using the 2^−ΔΔCt^ method (Livak and Schmittgen, 2001[23]). Table 1(Tab. 1) lists the primer sequences utilized in this study for real-time PCR analysis.

### Statistical analysis

The Statistical Package for the Social Sciences was used to analyze the data (ver. 22.0; SPSS Incorporated, Chicago, IL, USA). Normally distributed biochemical variables were presented as mean ± standard deviation (SD). The quantitative data was evaluated by the independent-samples t-test, and one-way ANOVA test. Quantitative data were shown as mean ± SEM using GraphPad Prism software version 8.4.3 (GraphPad Software, La Jolla, CA). The Pearson correlation coefficient was utilized to analyze the correlation between parameters for parametric data. A *P*-value of less than 0.05 was defined as statistically significant.

## Results

### Patients' basic features and laboratory examinations

Age, gender, BMI, DBP, SBP and FBS did not significantly between the control and CAD Groups. LDL, TC, TG and CPK-MB were significantly higher in the CAD group than in the control group. Also, HDL was significantly higher in the control group than in the CAD group (Table 2(Tab. 2)).

### Expression levels of CCL3, CCL4, IL-1β, TNFαIP3, and NF-κBIα genes in participants

**Figure 1:**
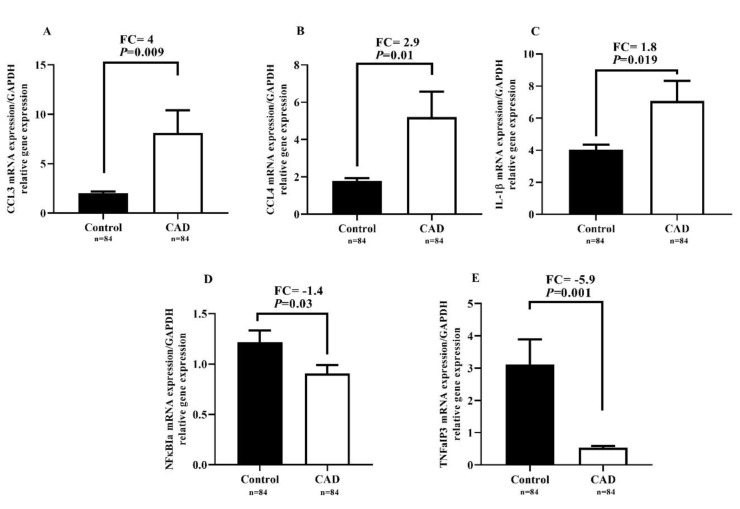
Relative expression levels of *CCL3*, *CCL4*, *IL-1β*, *TNFαIP3*, and *NF-κBIα* genes in CAD and control groups. *P*-values ≤0.05 were considered as significant using independent-samples t-test.

As shown in Figure 1A-C(Fig. 1), *CCL3*, *CCL4*, and *IL-1β *expression was significantly upregulated (FC=4, *P*=0.009; FC=2.9, *P*=0.01; FC=1.8, *P*=0.019, respectively) when comparing the CAD group with the control group. In contrast, *NF-κBIα* and *TNFαIP3 *expression was significantly downregulated (FC=-1.4, *P*=0.03 and FC=-5.9, *P*=0.001, respectively) in the CAD group compared to the control group, as shown in Figure 1D and 1E(Fig. 1).

### Expression levels of CCL3, CCL4, IL-1β, TNFαIP3, and NF-κBIα genes of the participants are associated with the number of clogged arteries

**Figure 2:**
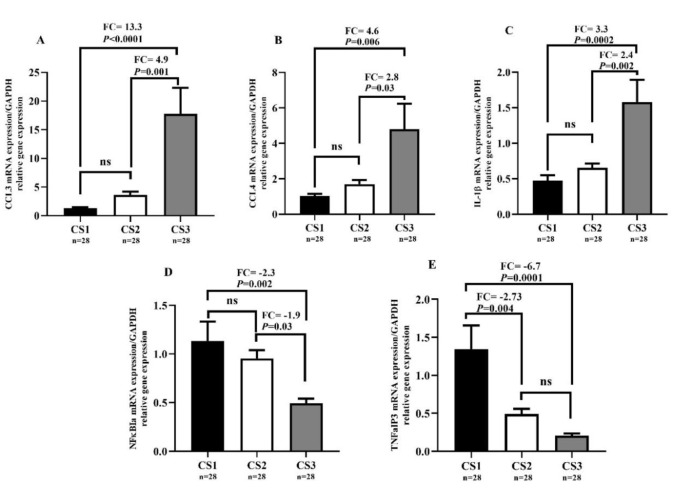
Relative expression levels of *CCL3*, *CCL4*, *IL-1β*, *TNFαIP3*, and *NF-κBIα* genes of the participants and their relation with the number of clogged arteries. *P*-values ≤0.05 were considered as significant using one-way ANOVA test and Tukey post hoc test multiple comparison analysis. Results are expressed as mean ± SEM.

According to the cardiologist's medical report, subjects with obstructive CAD were divided into three groups, i.e., obstructive CAD with cardiac arterial stenosis in one, two, and three main vessels were considered as CS1, CS2, and CS3, respectively. Tukey test multiple comparison analysis showed that *CCL3* expression was significantly upregulated in group CS3 compared to the groups CS1 and CS2 (FC=13.3, *P*<0.0001 and FC=4.9, *P*=0.001, respectively) as shown in Figure 2A(Fig. 2). In addition, no significant differences were found in *CCL3* expression between groups CS1 and CS2. *CCL4* expression was significantly upregulated in group CS3 compared to the groups CS1 and CS2 (FC=4.6, *P*=0.006 and FC=2.8, *P*=0.03, respectively), as shown in Figure 2B(Fig. 2). Moreover, no significant differences were found in *CCL4* expression between groups CS1 and CS2. As shown in Figure 2C(Fig. 2), *IL-1β* expression was significantly upregulated in group CS3 compared to the groups CS1 and CS2 (FC=3.3, *P*=0.0002 and FC=2.4, *P*=0.002, respectively). No significant differences were either found in *IL-1β* expression between CS1 and CS2 groups. In contrast, *NF-κBIα *expression was significantly downregulated in group CS3 compared to the group CS1 (FC=-2.3, *P*=0.002) and also in group CS3 compared to the group CS2 (FC=-1.9, *P*=0.03) as shown in Figure 2D(Fig. 2). No remarkable differences were found in *NF-κBIα* expression between groups CS2 and CS1. Finally, Figure 2E(Fig. 2) shows that *TNFαIP3* expression was significantly downregulated in group CS1 compared to the CS3 and CS2 groups (FC=-6.7, *P*=0.0001 and FC=-2.7, *P*=0.004, respectively). Besides, no significant differences were found in *TNFαIP3* expression between CS2 and CS3 groups.

### Expression levels of miR-24-3p and miR-595 in PBMCs of the participants are associated with the number of clogged arteries

**Figure 3:**
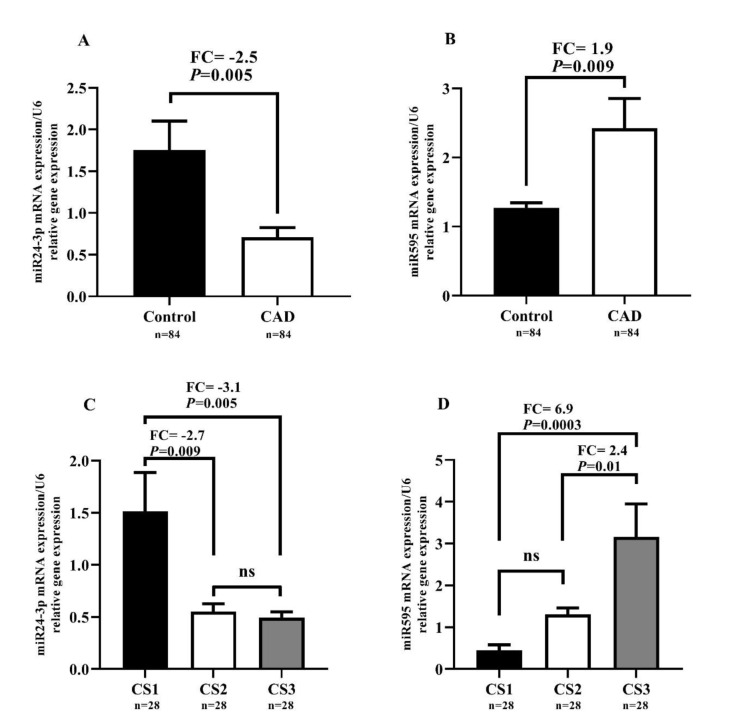
Relative expression levels of miR-24-3p and miR-595 in PBMCs of the participants and their relation with the number of clogged arteries. *P*-values ≤0.05 were considered as significant using independent-samples t-test, one-way ANOVA test (Tukey post hoc test) multiple comparison analysis. Results are expressed as the mean ± SEM.

As shown in Figure 3A(Fig. 3), miR-24-3p expression was significantly diminished (FC=-2.5, P=0.005) in the CAD group compared to the control group. In contrast, Figure 3B(Fig. 3) demonstrates that miR-595 expression was significantly enhanced (FC=1.9, *P*=0.009) when comparing the CAD group to the control group. As shown in Figure 3C(Fig. 3), miR-24-3p expression was significantly diminished in the groups CS2 and CS3 compared to the group CS1 (FC=-2.7, *P*=0.009 and FC=-3.1, *P*=0.005, respectively). No significant differences were found in miR-24-3p expression between groups CS2 and CS3. Meanwhile, miR-595 expression was considerably enhanced in group CS3 compared to the groups CS1 and CS2 (FC=6.9, *P*=0.0003 and FC=2.4, *P*=0.01, respectively), as shown in Figure 3D(Fig. 3). There was no difference in miR-595 expression between the groups CS2 and CS1.

### Correlation between expression levels of CCL3, CCL4, IL-1β, NF-κBIα, and TNFαIP3 genes in CAD group

The results of the correlation between the expression levels of *CCL3*, *CCL4*, *IL-1β*, *NF-κBIα*, *TNFαIP3* in the CAD group are presented in Table 3(Tab. 3). The expression levels of *CCL3* gene had a positive and significant correlation with *CCL4* and *IL-1β* expression (r = 0.404, *P*=0.005; r = 0.71, *P*<0.0001). The expression levels of *CCL3* gene had a negative and significant correlation with *NF-κBIα *and *TNFαIP3 *gene expression (r = -0.361, *P*=0.014 and r = -0.339, *P*=0.022, respectively). Moreover, the expression levels of *CCL4* gene had a positive and significant correlation with *IL-1β* expression (r =0.448, *P*=0.001). The expression levels of *CCL4* gene had a negative and significant correlation with *NF-κBIα *and *TNFαIP3 *expression (r = -0.457, *P*=0.001 and r = -0.385, *P*=0.009, respectively). The expression levels of *IL-1β* gene had a negative and significant correlation with *NF-κBIα *and *TNFαIP3 *gene expression (r =-0.367, *P*=0.017 and r = -0.397, *P*=0.005, respectively). On the other hand, the expression levels of *NF-κBIα* gene had a positive and significant correlation with *TNFαIP3* expression (r = 0.599, *P*<0.0001).

### Correlation between expression levels of miR-24-3p, and miR-595 with CCL3, CCL4, IL-1β, NF-κBIα, TNFαIP3 gene expression in the CAD group

The results of the correlation between the expression levels of miR-24-3p, and miR-595 with* CCL3*, *CCL4*, *IL-1β*, *NF-κBIα*, *TNFαIP3 *gene expression in the CAD group are presented in Table 3(Tab. 3). The expression levels of miR-24-3p had a negative and significant correlation with *CCL3*, *CCL4* and *IL-1β* gene expression (r = -0.337, *P*=0.023; r = -0.314, *P*=0.035; r = -0.407, *P*=0.005, respectively). The expression level of miR-24-3p had a positive and significant correlation with *NF-κBIα *and *TNFαIP3* gene expression (r = 0.426, *P*=0.003 and r = 0.412, *P*=0.005, respectively). The expression levels of miR-595 had also a positive and significant correlation with *CCL3*, *CCL4*, and *IL-1β* gene expression (r = 0.383, *P*=0.009; r = 0.475, *P*=0.001; r = 0.584, *P*<0.0001, respectively). The expression levels of miR-595 had a negative and significant correlation with *NF-κBIα *and* TNFαIP3* gene expression (r = -0.426, *P*=0.003 and r = 0.412, *P*=0.005, respectively). 

See also the Supplementary data.

## Discussion

The obtained results in the present study revealed that *CCL3* and *CCL4* expression levels were significantly higher in the CAD group compared to the control group. Furthermore, the higher grades of arterial stenosis in the CAD group were associated with higher levels of *CCL3* and *CCL4* gene expression. It was also found that* TNFαIP3* and *NF-κBIα* expression levels were considerably lower when comparing the CAD group to the control group, and higher grades of arterial stenosis were attributed to lower *TNFαIP3* and *NF-κBIα* gene expression. Besides, the expression level of *CCL3* gene had a positive and significant correlation with the expression level of *CCL4* gene, while the expression level of *CCL3* gene had a negative and significant correlation with the expression levels of *TNFαIP3 *and *NF-κBIα* genes in the CAD group. Moreover, the expression level of *CCL4* gene had a negative significant correlation with the expression levels of *TNFαIP3* and *NF-κBIα* genes in the CAD group. On the other hand, the expression level of *NF-κBIα* gene had a positive and significant correlation with that of the *TNFαIP3* gene in the CAD group.

A recent study into the effect of *CCL4* inhibition on the progression of atherosclerosis in ApoE knockout mice showed that *CCL4* inhibition reduced the atheroma areas and altered the development of atheroma plaques, resulting in a thicker fibrous cap, lower macrophage content, and lower matrix metalloproteinase (MMP)-2 and -9 expressions, implying plaque stabilization (Chang et al., 2020[3]). Another study aimed at elucidating the function of leukocyte-derived *CCL3* in atherogenesis found that in low-density lipoprotein receptor (LDLR^)-/-^ mice, under acute inflammation, leukocyte-derived *CCL3* might induce neutrophil chemotaxis against the atherosclerotic plaque, thereby accelerating lesion formation (de Jager et al., 2013[7]). A study aimed at determining the function of the *NF-κB* *inhibitor alpha* *(IκBα*) in atherosclerosis discovered that *IκBα*-knockout in myeloid cells of LDL-R-deficient mice promoted atherogenesis, most likely through induced leukocyte recruitment to plaques. IκBα-deficient macrophages had increased adhesion to an *in vitro* endothelial cell layer, which coincided with increased expression of the chemokine *CCL5* (Goossens et al., 2011[14]). Teplyakov et al. confirmed that initial cytokine concentration measurements revealed a high level of proinflammatory cytokines IL-1 (primarily *IL-1β*). In comparison, the shear stress probe, *IL-1α*, induced excessive *IL-1β* secretion, far exceeding the initial level and response to coagulation in atherosclerosis (Teplyakov et al., 2000[35]). Hasdai et al. reported that patients with ischemic heart disease, especially those with minimal CAD and angina, had higher serum *IL-1β* concentrations compared to the controls (Hasdai et al., 1996[18]). Moreover, the CANTOS (Cardiovascular Risk Reduction Study [Reduction in Recurrent Major CV Disease Events]; NCT01327846) trial using anti-inflammatory therapy with Canakinumab (anti-*IL-1β*) for atherosclerotic diseases recently demonstrated that reducing inflammation by IL-1β inhibition improved CVD outcomes (Ridker et al., 2017[33]). Furthermore, according to Wolfrum et al., in an analysis of the protective effect of *TNFαIP3* (A20) on atherosclerosis in apolipoprotein E-deficient mice, *A20* reduced atherosclerosis by lowering the *NF-κB* activity through reducing the expression of *NF-κB* target genes, thereby modulating the proinflammatory condition associated with lesion growth (Wolfrum et al., 2007[39]). Patel et al. demonstrated that the expression of *A20* in a SMC inhibited *NF‐κB* activation upstream of *IκBα* degradation that resulted in subsequent inhibition of NF‐κB‐dependent, proinflammatory, and proatherogenic proteins, such as CCL2 and Intercellular Adhesion Molecule 1 (ICAM-1). It prefers that *A20* is part of the physiological response of SMC to inflammation (Patel et al., 2006[29]).

Individual miRNAs can influence hundreds of gene transcripts to organize complex gene expression programs and, as a result, influence global changes in a cell's physiology. MiRNAs have thus been implicated as a key molecular player in almost every cellular phase, including cardiovascular development and pathophysiology (Eulalio et al., 2008[10]; Selbach et al., 2008[34]). The present study showed that miR-24-3p expression was significantly downregulated when comparing the CAD group to the control group and negatively correlated with the grade of arterial stenosis. It was also found that miR-595 expression was significantly upregulated when comparing the CAD group to the control group and was positively correlated with grade of arterial stenosis. In addition, a significant negative correlation was found between expression levels of miR-24-3p and expression levels of *CCL3* and *CCL4* genes in the CAD group. The expression levels of miR-595 had also a negative and significant correlation with the expression levels of *NF-κBIα* and *TNFαIP3* genes in the CAD group.

Similar to the findings of the present study, Zhu et al. reported that miR-24-3p was downregulated in arteriosclerotic arteries compared to normal arteries using quantitative real-time PCR and in-situ hybridization, implying that miR-24-3p regulated the proliferation and migration of human arterial smooth muscle cells (HASMC) by targeting platelet-derived growth factor receptor B (PDGFRB) and cellular myelocytomatosis oncogene (c-Myc) (Zhu et al., 2015[40]). Using a high throughput quantitative PCR technique to examine the expression changes of 40 different miRs in atherosclerosis, Gorur et al. concluded that miR-24-3p was reduced in atherosclerosis (Gorur et al., 2019[15]). Another study by Dang et al. using bioinformatics analysis and microarray in PBMC cells of patients with chronic obstructive pulmonary disease (COPD) showed that miR-24-3p regulated *CCL3*, *CCL4*, *IL-1β*, and *TNFαIP3* genes (Dang et al., 2017[6]). Unlike other results, Dolz et al., using a real-time PCR approach, reported that miR-24-3p had substantially higher expression in patients with asymptomatic carotid artery stenosis progression (Dolz et al., 2017[9]). The miR-595 was also found to be downregulated in hepatocellular carcinoma (HCC) tissues and cells, inhibiting cell proliferation, migration, and invasion *in vitro*. Furthermore, upregulating miR-595 expression in HCC cells inhibited the *NFκB* signaling pathway (Wang et al., 2020[38]). Another study reported that miR-595 expression was upregulated in malignant mesothelium, indicating that miR-595 might play a role in the development of this cancer (Guled et al., 2009[16]). In line with this finding, Hao et al. reported that miR-595 was frequently upregulated in clinical tissues and cells of glioblastoma multiform (GBM). Overexpression of miR-595 increased cell proliferation and growth of GBM cells, whereas depletion of miR-595 suppressed GBM cell growth and proliferation (Hao et al., 2016[17]).

In conclusion, our study demonstrated that decreased expression of miR-24-3p and increased expression of miR-595 in patients with CAD may lead to progression of atherosclerosis plaque by regulating the expression of *CCL3*, *CCL4*, *IL-1β*, *TNFαIP3*, and *NF-κBIα* genes.

## Declaration

### Acknowledgments

The authors are grateful to the staff of the Cellular and Molecular Research Center, Shahrekord University of Medical Sciences, and the authorities of the Angiography unit of Shahrekord Hajar Hospital for their valuable help.

### Statement of ethics

The Ethics Committee of Shahrekord University of Medical Sciences approved this study (IR.SKUMS.REC.1399.174). Written informed consent to participate in this study was obtained from all participants or their legal guardian before data collection.

### Conflict of interest statement

The authors have no conflicts of interest to declare.

### Funding sources

Shahrekord University of Medical Sciences financially supported this study with grant number 3118.

## Supplementary Material

Supplementary data

## Figures and Tables

**Table 1:**
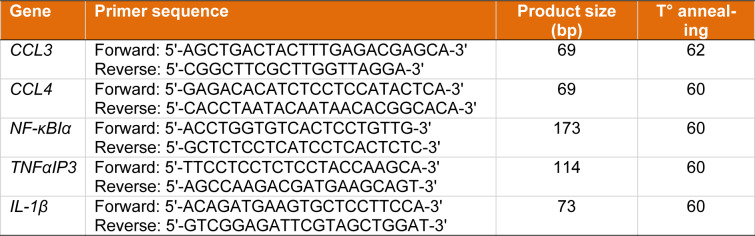
Primers used for real-time-PCR analysis

**Table 2:**
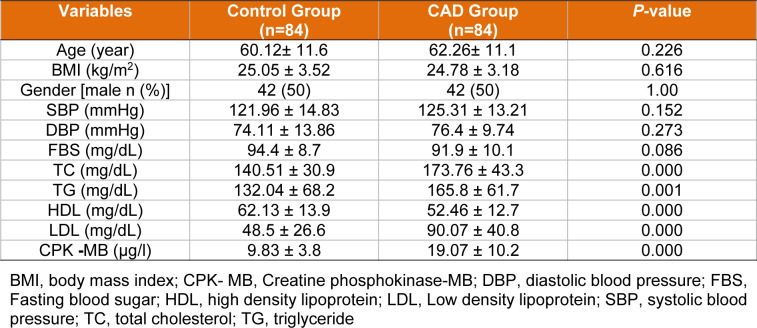
Anthropometric characteristics and biochemical parameters of the study subjects

**Table 3:**
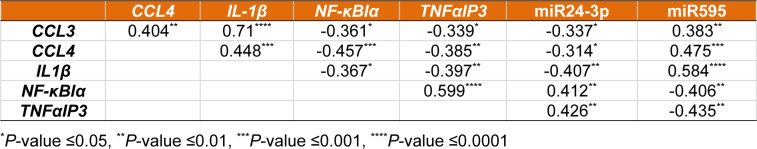
Pearson correlation between the levels of the different parameters in CAD group
